# Online education in palliative care - A national exploratory multimethod study

**DOI:** 10.1186/s12904-024-01615-z

**Published:** 2024-12-16

**Authors:** Carina Lundh Hagelin, Christina Melin-Johansson, Jane Österlind, Birgitta Bisholt, Susanna Pusa

**Affiliations:** 1 Department of Neurobiology, Care Sciences and Society, Division of Nursing, Karolinska Institutet, Stockholm, 171 77 Sweden; 2 Department of Health Care Sciences, Marie Cederschiöld University, Stockholm, Box 11189, 100 61 Sweden; 3 Department of Nursing, MidSweden University, Östersund, 831 25 Sweden; 4 Department of Healthcare Sciences, Swedish Red Cross University, Huddinge, Box 1059, 141 57 Sweden; 5 Department of Nursing, Umeå University, Umeå, 901 87 Sweden

**Keywords:** Continuous education, Distance learning, End-of-life, Health education, Nursing, Online learning, Palliative care

## Abstract

**Background:**

With an increased number of people living with multiple progressive diseases, online education courses have been created to address the growing need for competence in palliative care. However, there is limited knowledge about the form and content of these courses, or of participants’ experiences. This study aims to map the status, content, and evaluation of online palliative care courses in Sweden.

**Methods:**

This exploratory study used both quantitative and qualitative methods. The study process involved searching for online palliative care courses on the web and through contact with Swedish palliative care organisations, and then participating in these courses, surveying education providers, and analysing and validating responses. Quantitative data were analysed using descriptive statistics, while thematic analysis was applied to the free-text responses.

**Results:**

Nine online courses provided by five different organisations were mapped. These courses educated over 30 000 healthcare professionals, predominantly assistant nurses and registered nurses. There was a large discrepancy between the number of people who enrolled in and the number who completed the online courses. Shortcomings identified related to lack of systematic evaluation from the participants’ perspectives, if and how knowledge was integrated into clinical practice, and difficulties in making the courses sustainable.

**Conclusion:**

Overarching and national systems for online education are needed. These would require sustainability considerations and guidelines for implementation, evaluation and follow-up of non-university-based online educational initiatives in palliative care. In addition, it is crucial for employers to support professionals undergoing such education, ensuring that they are given opportunities to share their feelings and discuss any challenging thoughts that arise during and after the course.

**Supplementary Information:**

The online version contains supplementary material available at 10.1186/s12904-024-01615-z.

## Introduction

People worldwide are living longer and many will suffer from multiple progressive diseases associated with ageing, such as cancer, chronic heart failure, stroke, and dementia [1]. The World Health Organization (WHO) [2] estimates that nearly 57 million people globally need palliative care each year and that this number will increase significantly by 2060. Palliative care is also explicitly recognised as a human right to health [2]. There is, therefore, an urgent need to increase knowledge about palliative care among healthcare and social service professionals, as well as in society in general. It has been stated by the Council of Europe [3] and the World Health Assembly [4] that access to palliative care is a governmental responsibility; however, there is still unequal access to, and lack of knowledge about, palliative care [5–7]. All professionals involved in the care of people living with a serious life limiting disease, as well as the patients’ families, should have the skills to provide generalist palliative care [8]. This competence should include areas such as symptom management, team work, communication, and support for patients and families [9–12]. Different forms of teaching methods are often used for training in palliative care, such as lectures, seminars, and films [13, 14]. However, to be able to fulfill the coming requirement for palliative care, such education needs to be further developed [14]. This includes, for example, in areas of competence in palliative care in health and social care [8], competence in individual palliative care to achieve a person-centred approach, and for systematic competence development in palliative care, at all levels [8]. 

Palliative care is challenging since it is associated with end-of-life care and elicits different emotions for the patient, family members, and professionals. Caring for a seriously ill and dying person is complex and places great demands on nursing and medical professionals [15]. Quality-assured palliative care can ensure good care for patients, as well as security and safety for all parties involved [9]. 

In Sweden, the responsibility for the delivery of palliative care lies with the government, the regions, and municipal healthcare, ranging from specialist inpatient units to generalist palliative care services. The care can be provided outside of inpatient and hospital settings, for example in a patient’s own home or in an assisted living facility such as a nursing home. The healthcare professionals in the team around the patients vary in the different palliative care contexts. Assistant nurses are the largest group of professionals addressing the palliative care needs of the growing number of older people in nursing homes; [16] they are often supported outside of office hours only by an on-call registered nurse (RN). Contact with physicians and other team members is usually only on a consultative basis or at regular intervals. Assistant nurses have different professional titles in different countries, i.e., nurse assistant, enrolled nurse, or nurse aides. In Sweden, this profession is now a protected professional title, and in this study we use the term assistant nurse.

There is growing interest in, and an urgent need for, education in palliative care [7–14, 17]. As a result, alternative strategies for education, such as online courses, have been developed [14, 18]. Positive outcomes of such strategies include economic benefits, since they often reduce many of the costs associated with traditional classroom settings, and the flexibility they offer, allowing learners to study at their own pace and schedule [19]. However, there are also challenging aspects to consider, such as social detachment, which can lead to feelings of isolation among learners due to the lack of face-to-face interaction. Additionally, the need for clear instructions is crucial, as miscommunication can easily occur in an online environment, potentially hindering the learning process [19]. Online courses, currently accessed via the internet and found on the web, seem to differ in content and the amount of time required, and it is difficult to assess the content and quality of a course in advance. More knowledge about online education is needed, since its form and content can affect learning in both positive and negative ways [19]. The aim of this exploratory study was, therefore, to map the status, content, and evaluation of online palliative care courses in Sweden.

## Methods

**Fig. 1:**
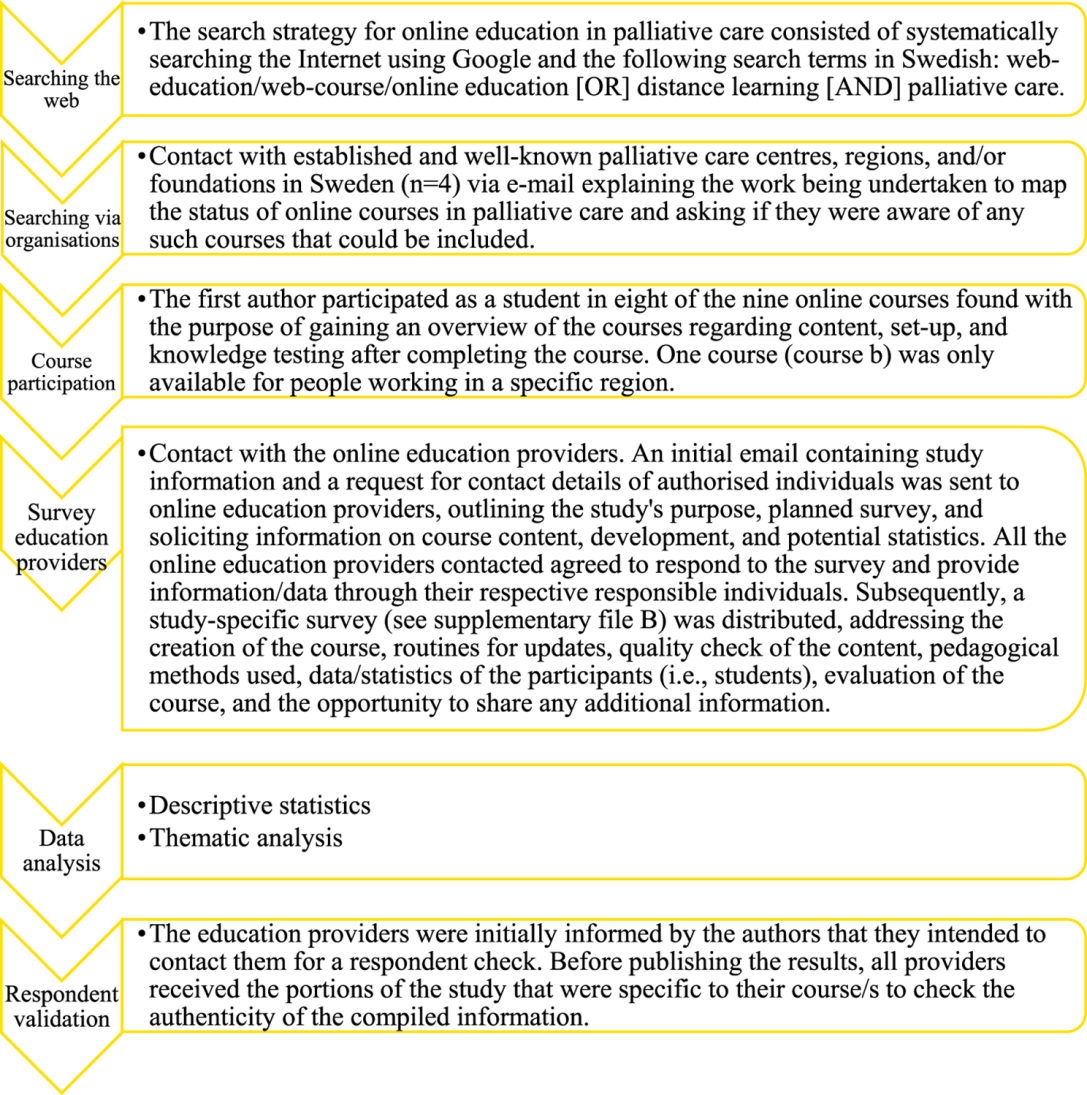
The steps used for data collection and data processing

The present study included both quantitative and qualitative methods comprising six steps: searching for online courses in palliative care on the web; contact with Swedish organisations associated with palliative care regarding courses; participation in courses; surveying organisations acting as education providers (Supplementary Material 2); data analysis; and respondent validation, Fig. 1. The inclusion criterion was open online courses with a primary focus on palliative care in Sweden. The exclusion criteria were courses at universities, blended learning, in-house training not for public use, online courses only developed because of the covid-19 pandemic, education courses with a fee, and courses available solely during specific dates. The research team consists of registered nurses with experience from both clinical palliative care and education and research on palliative care, and with good knowledge of the national organisations providing online education in palliative care. Standards for Quality Improvement Reporting Excellence in Education (SQUIRE-EDU) [20] were followed as a reporting guideline (see supplementary file A).

Quantitative data from the online education providers are presented using descriptive statistics. One provider continuously evaluated the participants’ perceptions of the specific online course (course i, Table 1), shared with the researchers both qualitative and quantitative data from an evaluation form given to participants to complete voluntarily. The evaluation form used by provider i consisted of 10 questions which had been answered by a total of 155 participants. The first four questions covered the participant’s characteristics, Table 3, and the next four questions covered the perceptions and experiences of the course, including free-text answers, Table 4. The last two questions allowed free text responses by participants on their overall perceptions and experiences (“what are your overall thoughts regarding the course” and “other comments”). Data were analysed using descriptive statistics and thematic analysis [21]. 

**Table 1:** Overview of the online courses in palliative care identified

Course	Target group	Goal	Time required (minutes)	Eligibility requirements	Certificate	Quality review	Evaluation Collection of participants’ data / participant evaluation of course
**a**	All healthcare professions who care for patients with PC needs	Increase competence among professionals working with patients where PC needs occur, regardless of workplace or profession. A sub-goal: increase interest in general PC among healthcare managers, which could in turn lead to local improvement work and an interest in the employees’ level of knowledge.	270	None	Yes, after the knowledge test	Yes	Yes / Partly* *Occasionally sent out a questionnaire and had individual conversations with managers and participants
**b**	All healthcare professions who meet patients and relatives at different stages of PC.	Increase competence in general PC with a focus on the PC approach, symptom relief, nursing, structured work methods and support of relatives.	270	Employed in the region	Not described	Yes	Yes / Partly* *At a specific time period 2017
**c**	Nursing professions	Increase knowledge and understanding and thereby support the employees in their meetings with patients in different PC stages and their relatives.	90	None	Yes, after the knowledge test	Yes	Yes / No
**d**	Nursing professions	Increase knowledge and understanding of symptoms and symptom relief in PC with the aim of increasing the quality of life of patients in different PC stages.	90	None	Yes, after the knowledge test	Yes	Yes / No
**e**	Primarily healthcare professions employed at nursing homes	Concrete suggestions about how to provide support to people in existential crisis and with a special section focusing on existential crisis in people with dementia and their relatives.	Not described	None	Yes, after the knowledge test	Yes	Yes /No
**f**	Nurses and physicians in specialised PC	Increase knowledge related to pain and relevant actions to be taken by healthcare professionals, together with the team, with the purpose of relieving pain. Increased security and safety in these matters among employees and the organisations.	Not described	None	Yes, after the knowledge test	Yes	Yes / No
**g**	Primarily assistant nurses	Provide a basic understanding of PC and what is important in encounters with elderly people approaching the end of life.	40	None	Yes, after the knowledge test	Unclear	? / ?
**h**	Assistant nurses, registered nurses, physicians, general practitioners in home healthcare	Ability to assess nutrition-based actions relevant in various situations and connected to each profession’s area of responsibility. Understand the importance of interprofessional collaboration and coordination of care interventions.	60	None	Yes, after the knowledge test	Yes	? / ?
**i**	All healthcare professionals	That all professionals within the team gain common basic knowledge to be able to perform good PC in the final stage of life.	210	None	Yes, after the knowledge test	Yes	Yes / Yes

**Table 2:** Overview of participants in respective online courses

Online course	Time period when data was collected	Number of participants who started the course	Number of participants who completed the course % (***n***)	Profession % (***n***)	
**a**	2014 (April) -2021 (May)	5561	53 (3835)	Registered nurse Assistant nurse Physician Allied health professional Counsellor Other	44 (2435) 43 (2403) 8 (472) 3 (176) < 1 (35) < 1 (40)
**b**	2016	~ 4000	-	-	
**c**	2017 (October) – 2021 (February)	2428	66 (1592)	Assistant nurse Registered nurse Other Physician Manager Physiotherapist Counsellor Administrator Researcher Dietitian	43 (1047) 32 (765) 9 (214) 9 (213) 2 (47) 2 (45) < 1 (22) < 1 (15) < 1 (5) < 1 (4)
**d**	2017 (October) – 2021 (February)	1629	76 (1238)	Assistant nurse Registered nurse Physician Other Physiotherapist Manager Administrator Counsellor Researcher Dietitian	45 (737) 29 (477) 10 (166) 9 (139) 2 (25) 1 (24) < 1 (12) < 1 (11) < 1 (5) < 1 (1)
**e**	n.d – 2021 (May)	1202	-	-	
**f**	n.d – 2021 (May)	2228	-	-	
**g**	-	-	-	-	
**h**	-	-	-	-	
**i**	2018–2021 (March)	38,776	59 (23063)	-	

- represents unknown data

**Table 3:** Participant characteristics (number of participants = 155)

Age (mean)	Place of work % (***n***)		Profession % (***n***)		Previous experience in PC % (***n***)	
20–71 (46)	Nursing home Municipal home care Home healthcare Student Hospital No workplace Specialist PC Other Missing	52.9 (82) 18.1 (28) 7.1 (11) 5.8 (9) 4.5 (7) 1.3 (2) 0.6 (1) 5.2 (8) 4.5 (7)	Assistant nurse Registered nurse Student Occupational therapist Physician Physiotherapist Manager Care assistant Teacher Counsellor Dietitian Other Missing	52.3 (81) 7.1 (11) 5.8 (9) 3.9 (6) 2.6 (4) 1.9 (3) 1.9 (3) 1.3 (2) 0.6 (1) 0.6 (1) 0.6 (1) 3.2 (5) 18 (28)	Yes No Missing	76 (118) 22 (34) 2 (3)

PC = Palliative Care

**Table 4:** Evaluation of the course (number of participants = 155)

Questions	Yes % (*n*)	No % (*n*)	Free-text answer	Missing
Was the course easily navigated?	79.4 (*n* = 123)	9.7 (*n* = 15)	7.1 (*n* = 11)	3.9 (*n* = 6)
Was the language easy to understand	89.0 (*n* = 138)	3.2 (*n* = 5)	2.6 (*n* = 4)	5.2 (*n* = 8)
Was the course at the right level for you?	78.7 (*n* = 122)	5.2 (*n* = 8)	10.3 (*n* = 16)	5.8 (*n* = 9)
Was the course relevant for you?	89.7 (*n* = 139)	3.2 (*n* = 5)	3.9 (*n* = 6)	3.2 (*n* = 5)

The process of the thematic analysis [21] involved moving back and forth between data, memos, codes, and themes. The analysis started with familiarisation with the data by reading all free text answers. Memos with potential codes were written down, and initial codes were created from data-driven meanings and patterns in the data. Codes were subsequently grouped into initial themes, which were then discussed and reviewed by all authors, resulting in rearranging codes, and merging, defining, and renaming themes. An inductive approach was applied where the identified themes had clear links to the data itself on a semantic level (i.e., the themes are identified within the explicit or surface meanings of the data).

## Results

### Findings are presented in two sections

*status and content of online courses in palliative care*, and *participant evaluation.* The first section consists of a presentation and description of the online courses, including a presentation of participants. The second section presents the findings from one online course (course i) that continuously evaluated the participants’ perceptions of the course.

### Status and content of online courses in palliative care

Nine online courses in palliative care were identified, developed, and operated by five different organisations, i.e., palliative centres, regions, and/or foundations, all free of charge for participants and either government funded or funded by a non-profit organisation. Six courses focused on general palliative care (courses a, b, c, d, g, i – see Table 1), and three on specific aspects of palliative care, e.g., existential issues (e), pain relief (f), nutrition (h), of which one focused on specialised palliative care (f).

Access to all the online courses was free of charge, Table 1, but for some, the participants needed to create an account to gain eligibility, and one of the courses (b) targeted only healthcare professionals in a specific region. Depending on the professional’s employment, they were either given access to the course via a learning platform or received an invitation link from their manager requiring login and a specific password.

The estimated time needed to complete the courses varied from 40 to 270 min, Table 1. The target groups for the courses were defined as either all healthcare professionals caring for patients with palliative care needs, nurses and physicians in specialised palliative care, or professionals in nursing homes or home care. However, the courses were also described as being useful for other professionals in the palliative care team, e.g., physiotherapists. Knowledge tests after completing the courses were common, comprising from 10 to 20 questions with a variation in degree of difficulty. Passing and obtaining a certificate required the participant to give correct answers to 80% of the questions. In some tests, the same questions were repeated, while others used randomly selected questions from a question bank and the tests could be performed several times.

All the courses had been checked by interprofessional groups, including physicians, RNs, assistant nurses and/or social workers. Two providers stated that they made revisions to their courses on an ongoing basis. There were differences with regard to whether a course was evaluated by the user after completion and the data that was collected. Some did not collect any data, some collected statistics concerning the number of people who had completed the course, while others collected and evaluated information concerning the profession of the participant, and the numbers who started and completed the course, Table 2. Available data showed that RNs and assistant nurses were in the clear majority (74–87%). About half of the online education providers reported that, in the last two years, they had reduced and finally closed their course since resources for continuous updates were lacking or not prioritised.

One online education provider highlighted the need for a national consensus concerning the content and structure of palliative care education. Available online courses were described as “creating a scattered education catalogue across the country, where providers have different views about the content and the recipients have no idea which course meets their needs”. A single national education package in general palliative care was seen to have the potential to reduce the risk of conceptual confusion, knowledge variations, and to reflect local or regional variations due to structures and organisation controlling the content. Through this, the education could cover palliative care independent of geography, age, gender, and diagnosis.

One provider stated that they continuously used surveys for evaluation of the course, both at the start and after completion of the course. Two other education providers reported that they had evaluated their course irregularly or during specific time periods.

### Participant evaluation

Analysis from the single education provider (i) that continuously collected evaluation data is presented below. The findings build on evaluations made between 2018 and 2021. In total, 23 063 persons completed the course, of whom 155 (0.67%) had answered the evaluation survey.

### Quantitative evaluation

Eleven different professions had answered the survey with more than half of participants being assistant nurses, followed by registered nurses, occupational therapists, and physicians. Most participants worked in nursing homes, municipal home care, or home care. Over three quarters of the participants had previous experience in palliative care. The characteristics of the participants are presented in Table 3. Most participants had perceived the course as being easy to navigate, using an understandable language, being at the right level and relevant, Table 4.

### Qualitative evaluation

Analysis of free text responses exhibited four themes: The set-up; Comprehending; Relevance; and Utilising.

#### The Set-up

includes participants’ perceptions of the content and structure of the online course. The form and structure, with self-study throughout, were perceived as suitable formats for learning. The time allowed to complete the course was considered too short by some who had spent more than the estimated time needed. Participants perceived that the online technique worked well overall, although some complications and technical problems were experienced, such as audio problems, navigation features not working, and problems with saving and returning to the right section. The flexibility, with options to choose how and where to attend the course, was appreciated. However, perceptions of being isolated in relation to the emotional impact of the subjects were described, and a need for discussion and reflection with others was mentioned.

*It was good but I don’t think it’s a course that’s suitable for self-study. I wanted contact with others for conversations during the course*,* I was emotionally affected and thought a lot about private things*. (no. 94)

Theoretical content in the course was perceived to be adequate and instructive, while the cases presented, and the palliative process were perceived as making it more understandable. Gender perspectives, age and religion were perceived to be reflected in the cases, but stereotypical gender roles existed where, for example, nurses were viewed as women and physicians as men. In addition, even though cultural aspects were acknowledged, an enhanced multicultural approach in the course material was requested.

#### Comprehending

consists of understanding the course, including the perceived level of the course, as well as aspects related to knowledge acquisition. In the comments on the level of the course, some described it as being at an appropriate level, others at a too advanced level, and some expressed a need for more in-depth knowledge. The provision of links and sources to more in-depth material was perceived as beneficial. The knowledge gained was seen as valuable for understanding one’s own abilities and to strengthen trust in one’s professional competence, even by those who had worked in palliative care for years.

*I have worked for many years in palliative care*,* but for me it was a good repetition and proof that I think in the right way.* (no. 95)

Participants’ perceptions of how easy it was to understand the content of the courses varied, with some experiencing it as being easy to understand while others expressed difficulties. Language barriers, such as not mastering the Swedish language fully in combination with some complex wording, were expressed. Presentations by different professionals in palliative care teams about their roles were perceived as supporting better comprehension of one’s own role, the roles and tasks of other professionals, as well as the relationships between different professional’s roles. This resulted in recognition of the value of the unique competence within healthcare teams. Taken together, participants expressed some variation in being able to comprehend and understand the content of the online course, including level, ease of use, and profession-specific values.

#### Relevance

includes the perceived relevance of the course concerning different professions, teamwork, and various healthcare settings. The importance of being relevant to different healthcare professionals and linking this to multi- and interprofessional teamwork was described. Perceptions regarding relevance, and the extent to which the professionals and contexts were depicted in relation to their own views, varied among participants.

The course was described overall as being applicable to multiple professionals and that “everyone involved in some kind of human care profession should complete it”. However, different healthcare professionals described not recognising their own professional role in palliative care. As one occupational therapist stated:

*I didn’t recognise myself at all in the depiction of what an occupational therapist does. // If one wants everyone in the team to attend the course and feel involved*,* it is important to highlight everyone and everyone’s work!* (no. 157)

The context (i.e., healthcare settings) included in the cases was described as too narrow and too in-patient focused. More variation regarding care environments and patient groups was requested. Having a team-based focus was perceived as ensuring the relevance of each professional’s importance in the team and the strength of teamwork in palliative care. Nevertheless, the perceptions of the amount of attention given to teamwork ranged from being sufficient to needing more clarity about how different professionals contribute to the team.

#### Utilising

includes participants’ perceived value of, and learning from, the online course. The focus was on knowledge gained and how to use it practically.

*I have updated my knowledge*,* helping me do an even better job in the future.* (no. 5)

The possibility for repetition and being able to go back, both during and after finishing the course, was described as supporting continuous professional development. Participants described a need for further knowledge about the palliative care approach and how to transform theoretical knowledge into practical skills through testing and practice. The course was described as reinforcing the importance of palliative care and that palliative care actions really matter.

## Discussion

Approximately 30 000 healthcare professionals had completed the nine online palliative care courses identified, which were developed and provided by five organisations from palliative care centres, regions, and/or foundations. This is many fewer than the 84 videos on YouTube identified by Liu et al. (2019) [22]; however, the courses we searched for and identified were from authorised providers and all but one were clearly quality reviewed. The content of courses varied, with topics primarily related to general palliative care, although with a more specific focus in some areas. It must be noted that only one provider systematically performed evaluation. A large proportion of healthcare professionals had completed the online courses, although some persons might have attended several courses. The large difference between the number of participants registered and those who completed the course might indicate that the set-up and methods used are not suitable for all and need to be further developed. A course evaluation should therefore be carried out to identify the reason for this.

The online courses were estimated to take between 40 min and 270 min to complete; however, more time was sometimes needed, contributing to difficulties in planning for completion. This is far longer than the YouTube videos [22], which lasted no more than 6 min. In addition, the structure and content of courses might also be more or less suited to different learning styles and learning abilities. The courses identified in this study seem to be easy to access and, apart from one course, not limited to geographical areas, which provides a valuable opportunity for different professionals to maintain and increase their knowledge. Several aspects of learning need to be included in the development of this type of course, such as the type of language used, methods of learning, and the creation of opportunities for in-depth discussions [8, 14, 22]. Further, an awareness of the use of stereotypical gender roles and normative cultures must be addressed [22].

The results from the present study support the knowledge that online education courses can be effective and work well [19, 23], which is represented by the total number of people participating in the courses. A reported facilitating factor was the possibility for repetition and to go back into the course, both during and after completion. However, the use of this possibility might be related to the subject matter in the course, that not everyone appreciates this type of education, that people participate alone or that the course does not fulfil the requirements. The identified open online courses addressed several of the core competence areas in palliative care [11], however other areas were not addressed, such as the challenges of clinical and ethical decision-making, the development of interpersonal and communication skills, practicing self-awareness, and continuing professional development. These competencies might be difficult to develop in online courses with no interaction with a teacher or fellow students.

Palliative care is often experienced as challenging and complex [15, 24–26] and an area that frequently evokes strong emotions [27, 28]. It is also difficult to both learn and teach, even in face-to-face education [13, 24]. It is not uncommon that there are many questions about the concepts of end-of-life care and of palliative care which need to be explained and clarified [29], as well as several aspects of the complexity of palliative care that are required to achieve quality [8, 14]. Occasions for reflection and active engagement with teachers and fellow learners were lacking in the online courses included, which worked for some participants but was more difficult for others. Integrating contacts with teachers or fellow students in some way would be beneficial to increase learning [30], but might be difficult to arrange in online education. This, together with the possibility that the learner may lose focus due to distraction from other things in the online course [30], makes follow-up important. When feedback is not provided throughout the training, employers must take responsibility for confirming, following-up and enabling reflection, which means that a large part of the responsibility for knowledge development must be placed on the employer. Despite the government being responsible for the palliative care provided in the country, there is currently no overarching organisation in Sweden controlling the content of the type of education that is considered in the present study, nor its follow-up or development [2, 3]. To meet the increased demand for competence in palliative care [1] it would be of value to have a national online education or framework that meets the needs for training of all professionals. The present study highlights aspects that need to be related to education in palliative care apart from core competencies [10–12], such as a variety of methods of learning, evaluation, and continuous course development.

Few educators evaluated the participant’s satisfaction with a course, which is in line with other reports [17]. In some courses, the participants received a certificate after passing a knowledge test, but no follow-up activities were performed. It is not known how an individual’s new knowledge is integrated into the clinical setting; employers therefore need to lead further reflection and training in the work context. High-quality online education in palliative care, suitable for different healthcare professionals, is important to consider, and there seems to be a need for a nationally agreed minimum level of education in general palliative care.

Authorities, politicians, decision-makers, educators, and organisations need to act as soon as possible to ensure access to standardised education to ensure that professionals have the appropriate skills [2, 3]. Online education can be effective; [23, 31] this is besides the need to integrate palliative care into undergraduate curricula [32]. However, learning interventions must be available for different learning styles, and the content of courses updated in line with the development of knowledge to meet the future needs of palliative care [8, 9, 13, 14]. This study does not provide comprehensive information about online palliative care education. Instead it provides knowledge about the overall status of online education courses in palliative care in Sweden and highlights some aspects that need to be considered by actors responsible for providing online courses nationally, as well as internationally.

### Methodological considerations

There is not one path for mapping online education, and we used a six-step approach, although some methodological considerations need to be discussed. A strength of this study is the use of both quantitative and qualitative methods. The expertise within the research group is also a strength, enabling the identification of the available online courses. On the other hand, if the courses are difficult to find by searching on the internet, they might not be achieving their purpose.

It can be argued that the survey was perhaps too broad. However, the survey questions were compiled to gain a broad overview of online courses in Sweden, which we regard as meaningful, and the data to map the different online courses included in the study. This is a first attempt to explore online courses regarding their status, content, and evaluation. Since there is no uniform way to evaluate or compile statistics from the online education providers, we think this study presents learning aspects for the development and evaluation of online palliative care courses.

There are differences in the ways the courses were evaluated by the users after completion. Thus, the data presented about participants starting and completing a course, and their professions, are not comprehensive. In addition, the overall numbers of healthcare professionals included should be understood as the summed numbers from all the online courses that collected data, i.e., not as the number of exclusive individuals. One author participated in the courses, which could be considered both a strength and a limitation. The author’s participation was valued as enhancing the understanding of content, set-up and knowledge tests, but could also have influenced the thematic analysis. However, throughout the qualitative thematic analysis, the researchers sought to minimise the influence of their pre-understanding. The authors acknowledge that the contextual content of palliative care, as well as their clinical-, pedagogical-, and scientific experience from the field, facilitated the set-up, analysis and reporting of the study. All steps in the analysis were discussed initially by the first and last author, followed by discussions in the whole research group to enhance trustworthiness. Moreover, in the analysis of the course (i) that continuously collected evaluation data, the findings are based on 155 of 23 063 participants who answered the optional evaluation after completing the online course. Thus, the very low response rate means the results should be viewed with caution. On the other hand, this contributes to the discussion regarding the urgent need for developing structures for quality assessment, participant evaluation, and feedback in existing and future online education in palliative care.

## Conclusion

Online education in palliative care seems to have the potential to reach professionals in different care settings and is a way to increase knowledge about general palliative care. However, there is a need for a national system to provide clear structures for implementation, evaluation, and follow-up of non-university-based palliative care education initiatives, and to provide financial opportunities for sustainable, adapted, and constantly updated online courses from a national perspective. Furthermore, it is important that employers recognise and enable the support of professionals undertaking this type of course during which they are not able to share their feelings or discuss difficult thoughts with anyone. There is, to the best of our knowledge, no existing similar study, so we cannot compare our results with online initiatives from other countries. Based on the results of this study, analyses conducted in other countries could contribute to the development of knowledge about online courses in palliative care. In addition, further research is needed on the ways of conducting online training, communication, sharing training experiences, personalised development through training, digital simulation, and how education can provide a higher quality of palliative care.

## Electronic supplementary material

Below is the link to the electronic supplementary material.

Supplementary Material 1

Supplementary Material 2

## Data Availability

Data is provided within the manuscript or supplementary information files.
