# Correction: Online education in palliative care—A national exploratory multimethod study

**DOI:** 10.1186/s12904-025-01662-0

**Published:** 2025-01-25

**Authors:** Carina Lundh Hagelin, Christina Melin-Johansson, Jane Österlind, Birgitta Bisholt, Susanna Pusa

**Affiliations:** 1 Department of Neurobiology, Care Sciences and Society, Division of Nursing, Karolinska Institutet, Stockholm, 171 77 Sweden; 2 Department of Health Care Sciences, Marie Cederschiöld University, Box 11189, Stockholm, 100 61 Sweden; 3 Department of Nursing, MidSweden University, Östersund, 831 25 Sweden; 4 Department of Healthcare Sciences, Swedish Red Cross University, Box 1059, Huddinge, 141 57 Sweden; 5 Department of Nursing, Umeå University, Umeå, 901 87 Sweden

**Correction: BMC Palliat Care 23, 283 (2024)**

**https://doi.org/10.1186/s12904-024-01615-z**

Following publication of the original article [1], the author reported that in Fig. 1, some texts were in red font when it should be black.

Old figure

**Figure 1:**
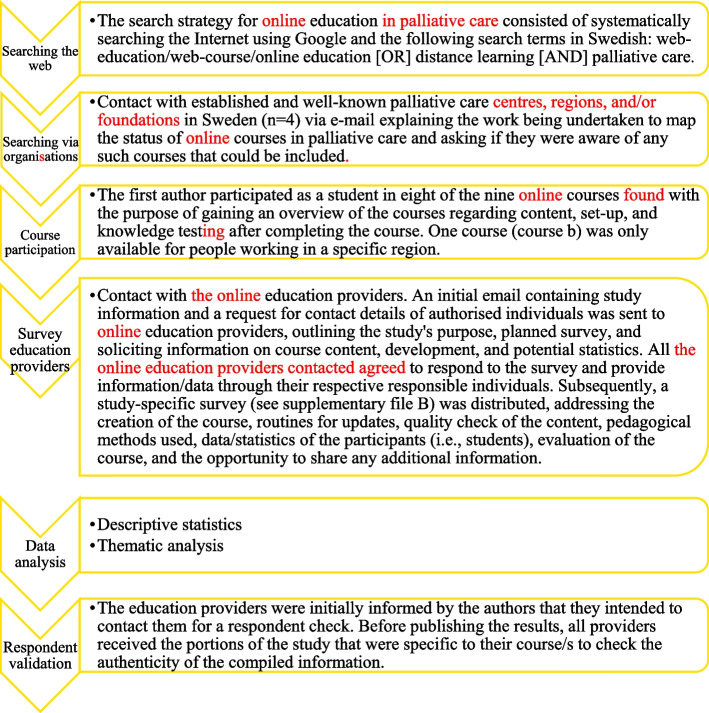

New Figure
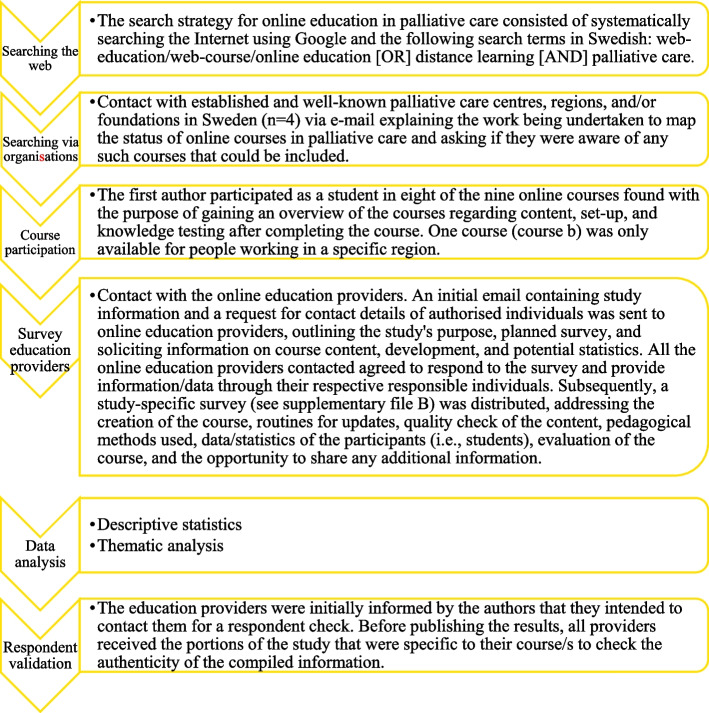


The original article has been updated.
