# Supplementary material for: Online education in palliative care - A national exploratory multimethod study
Source: BMC Palliat Care. 2024 Dec 16;23:283. doi: 10.1186/s12904-024-01615-z (PMC11650833; doi:10.1186/s12904-024-01615-z)
Supplement: Supplementary file 1 — Supplementary Material 1 [file 12904_2024_1615_MOESM1_ESM.docx]

**SQUIRE-EDU (Standards for QUality Improvement Reporting Excellence in Education)**

| ***No. Item*** | ***Guide questions/description*** | ***Page nr.*** |
| --- | --- | --- |
| **TITLE & ABSTRACT** | | |
| 1. Title | EDU 1: Indicate that the manuscript concerns efforts to improve health professions education systems and learning | p.1 |
| 2. Abstract | EDU 2: Keywords include a focus on education and learning | p.2 |
| **INTRODUCTION** | | |
| 3. Problem description | EDU 3: Description of the nature and significance of the need for change in the local educational system | p.3 |
| 4. Available knowledge | Summary of what is currently known about the problem, including relevant previous studies | p.3 |
| 5. Rationale | EDU 5: Identify the guiding theory (learning, change, implementation, or other) and how it aligns with the need for change in the local educational system | p.3 |
| 6. Specific aims | Purpose of the project and of this report | p.4 |
| **METHODS** | | |
| 7. Context | EDU 7a: Contextual elements for learning (e.g., setting, program, people, resources, social, geopolitical influences) before the intervention(s)  EDU 7b: The interrelationships between the contextual elements and the local educational and healthcare systems before the intervention(s) | p.4-5  N/A |
| 8. Intervention(s) | EDU 8a: Description of the primary interventions and cointerventions (e.g., faculty or tool development)  EDU 8b: Specify how the interprofessional education team (e.g., faculty, staff, patients, and learners) was part of the design of the intervention | This is a part of the findings, presented on p.6-8 + Table 1)  p.9 |
| 9. Study of the intervention(s) | EDU 9a: Approach used to understand the impact of the educational intervention(s) on the learner and beyond, such as impact on patients, families, the community, faculty, educational program, or the healthcare system  EDU 9b: Approach to assess the fidelity of and the iterative changes to the planned intervention(s) over time | This is a part of the findings, presented on p.12-14.  p. 8, Table 1. |
| 10. Measures | EDU 10: Quantitative and/or qualitative measures chosen to assess the educational processes and outcomes on learners, faculty, educational programs, patients, families, healthcare systems, or communities | p.5 + Figure 1. |
| 11. Analysis | a. Qualitative and quantitative methods used to draw inferences from the data  b. Methods for understanding variation within the data, including the effects of time as a variable | p.5-6 + Figure 1.  N/A |
| 12. Ethical considerations | EDU 12: Approaches to address vulnerability of learner participants | p.6, 17. |
| **RESULTS** | | |
| 13. Results | EDU 13a: For each educational intervention and cointervention, provide details about iterative modifications based on the assessment of the learning | p.9 (N/A) |
| **DISCUSSION** | | |
| 14. Summary | Connect the findings to the guiding theory (learning, change, implementation, other) used to direct the change in the local educational system | p.14-15 |
| 15. Interpretation | EDU 15c: Include the impact of the intervention(s) on learners, faculty, educational program, patients, families, healthcare systems, or communities | p.15-16 |
| 16. Limitations | a. Limits to the generalizability of the work b. Factors that might have limited internal validity such as confounding, bias, or imprecision in the design, methods, measurement, or analysis c. Efforts made to minimize and adjust for limitations | p.16 |
| 17. Conclusions | EDU 17b: Scalability of the work to other learners and contexts EDU 17d: Lessons learned for clinical practice, education, and policy | p.16-17 |
| **OTHER INFORMATION** | | |
| 18. Funding | Sources of funding that supported this work. Role, if any, of the funding organization in the design, implementation, interpretation, and reporting | p.17 |

Developed from: Ogrinc G, Armstrong GE, Dolansky MA, Singh MK, Davies L. SQUIRE-EDU (Standards for QUality Improvement Reporting Excellence in Education): Publication Guidelines for Educational Improvement. Acad Med. 2019;94(10):1461-1470.
