# Supplementary material for: Online education in palliative care - A national exploratory multimethod study
Source: BMC Palliat Care. 2024 Dec 16;23:283. doi: 10.1186/s12904-024-01615-z (PMC11650833; doi:10.1186/s12904-024-01615-z)
Supplement: Supplementary file 2 — Supplementary Material 2 [file 12904_2024_1615_MOESM2_ESM.docx]

Study-specific survey to education providers

**Q1**: How was this education created?

- Which year?
- What did the initiative look like?
- Was the education prompted by some form of needs assessment and/or was there a perceived need for the education?
- Any collaborations in the development of the education?
- What is the purpose of the education?
- What does the education rely on (e.g., approaches, definitions, theories)?
- Who is the education aimed at?
- What are the expected outcomes of the education (goals, etc.)?

**Q2**: Has the education been updated since its creation? (Y/N)

- If you answered "YES": Who is responsible for the updates?
- How are the updates carried out?

**Q3**: How has quality regarding the content of the education been managed and potentially ensured (e.g., fact-checking)?

**Q4**: Have you employed any pedagogical methods in creating the education, if so, in what way?

**Q5**: Are there any data/statistics available about the individuals who have completed the education (e.g., number and profession)? (Y/N)

- If you answered "YES": Is it possible for our research group to access this information to compile/report in the study?

**Q6**: Has the education been evaluated by the users?

- If you answered "YES": In what manner?
- Is it possible for our research group to access this information to compile/report in the study? (Y/N)

**Q7**: Is there anything else beyond the above questions that you would like to share regarding the current web-based education?
